# Characterization of the vastus lateralis torque-length, and knee extensors torque-velocity and power-velocity relationships in people with Parkinson's disease

**DOI:** 10.3389/fspor.2024.1380864

**Published:** 2024-04-25

**Authors:** Riccardo Magris, Francesca Nardello, Federica Bombieri, Andrea Monte, Paola Zamparo

**Affiliations:** Department of Neurosciences, Biomedicine and Movement Sciences, University of Verona, Verona, Italy

**Keywords:** force-velocity relationship, muscle disorders, muscle mechanics, muscle architecture, mechanical power

## Abstract

**Introduction:**

Parkinson's disease (PD) is a prevalent neurodegenerative condition observed primarily in the elderly population that gives rise to motor and non-motor symptoms, one of which is muscle weakness. The aim of this study was to characterize the vastus lateralis torque-fascicle length (T-L) and the knee extensors torque-angular velocity (T-V) and power-angular velocity (P-V) relationships in PD patients and to investigate the influence of muscle geometry on muscle mechanics.

**Methods:**

Participants (11 PD: patients, 9 CR: age matched healthy controls; 10 CY: young healthy controls) performed: (i) isometric contractions (e.g., MVC) to obtain the torque-angle and T-L relationships; (ii) isokinetic (e.g., iso-velocity) contractions to obtain the T-V and P-V relationships. During the experiments, the architecture of vastus lateralis (pennation angle, fascicle length, muscle thickness) was also determined by using an ultrasound apparatus.

**Results:**

Significant differences were observed between PD patients and physically matched control groups (CR and CY) in terms of maximum isometric force (calculated as the apex of the T-L curve) and maximum mechanical power (apex of the P-V curve), but not in maximum shortening velocity. Among the mechanical variables investigated, mechanical power was able to identify differences between the less and the more affected side in PD patients, suggesting that this parameter could be useful for clinical evaluation in this population.

**Conclusions:**

The observed results cannot be explained by differences in muscle geometry at rest (similar in the three cohorts), but rather by the muscle capacity to change in shape during contraction, that is impaired in PD patients.

## Introduction

The evaluation of the muscle's mechanical output by means of the torque-length (T-L), torque-angular velocity (T-V) and power-angular velocity (P-V) relationships has gained increasing attention in recent years due to their association with physical performance in populations ranging from young people (and athletes) to elderly people with or without pathological conditions (1, 2).

The T-V relationship illustrates the ability to produce torque at different contraction velocities and can be used to identify muscle weakness at different/specific torque or velocity levels; this allows for a better characterization of a subject's functional capacity during daily life activities (3) compared to standard isometric evaluations: the higher the torque produced at a given movement velocity, the higher the mechanical power generated. Mechanical power is negatively and independently associated with the risk of cognitive decline (4), mobility limitations, disability (5) and hospitalisation (6) in older adults. Therefore, the evaluation of the P-V relationship is also of scientific interest to better identify the loss of function in several pathologies. Last but not least, the T-L curve could also provide important information about people's physical fitness since it describes a muscle's capability to generate torque at any given fascicle length. Together, these three relationships could provide important insights into a specific population's muscle's mechanical capacity.

To our knowledge, a population that has never been characterised in terms of T-L, T-V and P-V relationships is that of subjects with Parkinson's disease (PD). Parkinson's disease is a prevalent neurodegenerative condition observed primarily in the elderly population, affecting approximately 1% of individuals aged 60 years and above (7). This pathological state gives rise to motor and non-motor symptoms, including bradykinesia, rigidity, tremor, and postural instability. These symptoms collectively contribute to a progressive deterioration of functional abilities, ultimately leading to disability. The decrease in functional performance observed in patients with Parkinson's disease (PD) can be attributed to various factors, one of which is muscle weakness (8). This weakness is linked to both central and peripheral alterations, such as a decrease in voluntary activation (9), altered spatial activation pattern (10), diminished fascicle shortening and tendon elongation (11), increased muscle stiffness (12) and decreased muscle's capability to change in shape (13). Although the effects of the central parameters in determining the loss of force in people with PD are well acknowledged in the literature [e.g., (14, 15)], the impact of peripheral variables remains unclear.

In this regard, from a neural/nervous point of view, a lower EMG amplitude during maximum explosive contractions was observed in PD patients compared to healthy age-matched control participants (13, 15). Furthermore, PD patients exhibit higher antagonist co-activation (16) and variable motor unit discharge rates (17) during maximal and submaximal isometric force reduction compared to healthy subjects, which in turn decrease force production capacity (18). From a peripheral point of view, alterations in muscle architecture could (at least partially) explain the observed loss of force (and hence of torque). For example, changes in muscle thickness and/or physiological cross-sectional area are related to a loss of maximum force in elderly people (19). On the other hand, decreases in pennation angle and fascicle length could be related to a decrease in muscle force and fascicle shortening velocity, respectively (20). These geometrical modifications could be associated to changes in the T-L, T-V and P-V relationships. For example, a decrease in fascicle length is related to a reduction in the maximum shortening velocity and a decrease in the fascicle operating length along its T-L relationship (21, 22).

Resting muscle geometry was observed to be similar between PD patients and age and physically-matched control groups (13, 15) whereas fascicle and muscle behaviour were found to differ in dynamic conditions (e.g., during explosive contractions; (13). Indeed, PD patients showed higher values of MTU stiffness during contraction compared to healthy controls reducing the muscle's capability to change in shape and to increase force production rapidly (13). Therefore, the concomitant evaluation of muscle geometry at rest and during contraction, as well as that of the torque-length and power-velocity relationships, could represent an important screening for people with PD, providing information about the level/progression of this pathology.

Therefore, this study's primary aim was to evaluate the possible changes in muscle geometry at rest and during contraction and to investigate how these changes could affect the T-L, T-V and P-V relationships in PD patients. Furthermore, we compared data obtained in this specific population with those obtained in age and physically-matched cohorts and a young control group to better distinguish the changes imposed by PD from those typically related to the ageing process.

We hypothesized no significant differences in resting muscle geometry between PD patients and the age and physically-matched group, as previously reported by Martignon et al. (15) and Monte et al. (13). However, significant differences between PD patients and control groups were expected in terms of architectural behavior during the characterization of the T-L, T-V, and P-V relationships (as previously found during explosive contractions by (13). We then expected that the differences in muscle fascicle behaviour exhibited by PD patients could affect these relationships (e.g., lower torque, angular velocity and power in PD patients). Finally, since the onset of pathological symptoms in PD exhibits asymmetry (resulting in a higher degree of impairment of one limb) we hypothesised that the torque, angular velocity and power were reduced in the more affected limb compared to the less affected one.

## Materials and methods

### Participants

In this study we calculated the sample size *a-priori* with an effect size of 0.55 (e.g., obtained from the literature based on the differences in maximum voluntary torque between PD patients and healthy controls (15);, an alfa-level of 0.05, a statistical power of 0.8 and three groups of subjects (PD, CR and CY). The total sample size was 36 (12 participants for each group). For this study, we recruited 12 patients with mild to moderate Parkinson's disease (PD), 16 healthy age-matched controls (CR), and 14 healthy young controls (CY).

For PD patients, the inclusion criteria were a diagnosis of idiopathic PD performed by a neurologist, according to the London Brain Bank guidelines. All subjects were characterized by postural instability and a gait disorders phenotype (PIDG). The disease severity was calculated based on the modified Hoehn and Yahr scale, and only subjects up to stage 3 were recruited. All procedures were conducted during the medication “on” condition of the dopaminergic treatment. For all participants (PD, CR and CY), exclusion criteria were any type of dementia, inability to walk and muscular injuries. Subjects with cardiovascular, orthopaedic, or metabolic disorders were also excluded from the cohorts.

All participants received written and oral information and instructions before the study and gave their written informed consent to the experimental procedure. The experimental protocol was approved by the Ethical Committee of the University of Verona (protocol number: 2021-UNVRCLE-0450152) and was performed in accordance with the Helsinki Declaration.

### Experimental design

Healthy subjects (CR and CY) participated in a single session during which only their dominant lower limb was tested whereas PD patients performed two sessions during which the more affected limb (PDA) and the less affected limb (PDNA) (as ranked by their neurologist) were both tested.

The entire protocol involved: (i) isometric contractions (e.g., maximum voluntary fixed-end contractions, MVC) to obtain the torque-angle and torque-fascicle length relationships; (ii) isokinetic (e.g., iso-velocity) contractions to obtain the torque-angular velocity and power-angular velocity relationships.

During the experiments, the vastus lateralis (VL) fascicles length was determined by using an ultrasound apparatus.

### Data collection

#### T-Angle, T-L, T-V and P-V relationships

The participants were secured on a dynamometer (100 Hz; Cybex NORM, Computer Sports Medicine Inc., Stoughton, USA) using a trunk and pelvic strap. They were seated with their hip fixed at 85° of flexion (23) with the arms crossed in front of the chest. The participant's lower leg was fixed with Velcro straps around the mid-shank to a cushioned attachment connected to the dynamometer's lever arm. The rotational axis of the dynamometer was carefully aligned with the axis of rotation of the knee joint during a maximal contraction at 60° knee flexion (24). A standardized warm-up for each contraction type was conducted to familiarise the subjects with the task (see Supplementary Figure S1). Firstly, to account for the effects of gravity and passive joint torque on the net knee joint torque, three passive knee extensions were performed over the knee joint's range of motion, while participants were instructed to relax (25). Secondly, the subjects were instructed to push “as hard as possible” for 3–4 s (for the MVCs) or over the entire ROM (i.e., from 90° to 0°) (for the iso-velocity trials).

Participants performed three consecutive MVCs (knee extensions) at six knee joint angles: 15, 30, 45, 60, 75 and 90° (where 0° = knee fully extended) and three consecutive iso-velocity knee extensions at five angular speeds: 45, 90, 150, 210 and 250°/s. For each iso-velocity trial, participants were instructed to extend their knee as hard as possible starting ∼0.5 s before the knee extension. This protocol pre-loaded the quadriceps muscles, facilitating maximal voluntary neuromuscular activation and maximum torque throughout the entire ROM (26, 27).

Between contractions, at least 2 min of rest were provided to minimize fatigue (28). A randomized order has been followed and the participants received strong verbal encouragement during each MVCs and each iso-velocity knee extension.

A 2D video analysis was utilized to verify the actual knee angle during all contractions based on the following markers: greater trochanter of the opposite side, the lower portion of the patella (patellar tendon origin), upper anterior surface of the tibia (patellar tendon insertion), knee center of rotation and medial malleolus. The marker positions were recorded by means of a Casio Exilim Camera (100 Hz, Casio Computer Co., Ltd., Tokyo, Japan) and analysed with a video processing software (Tracker v6.0.10). The camera was positioned on the opposite side of the subject, perpendicular to the thigh longitudinal axis.

During the MVCs, muscle architecture behaviour was recorded by means of a B-mode ultrasound apparatus (Telemed MicrUs EXT-1H rev.D, Lithuania) with a 6 cm linear array probe (with a depth and width of 60 and 60 mm, respectively) and a sampling frequency of 12 Hz. The probe was fixed to the skin by means of a plastic strap approximately at 50% of the femoral length, aligned on the muscle belly and corrected with respect to the superficial and deep aponeurosis to have a clear image of the perimysial connective intramuscular tissue, that it is indicative of the muscle fascicle structure. The probe was never removed during the entire experimental session. The muscle architecture parameters were calculated from the ultrasound videos (see Data Analysis below). All devices were synchronised with an external manual trigger (5 V) and data were collected by means of LabChart (V.6).

#### Questionnaires

Physical activity level was investigated by means of the International Physical Activity Questionnaire (29), while the cognitive function was assessed using the Mini Mental State Examination (MMSE) (30). The disease severity was classified according to the Hoehn and Yahr scale (31), while the assessment of the degree of motor and functional impairment was determined using part III of the Unified Parkinson's Disease Rating Scale (UPDRS) (32).

### Data analysis

The offline analysis was conducted using custom-developed programs in Matlab (r2021a).

#### T-Angle, T-L, T-V and P-V relationships

The total torque generated by the knee extensors was corrected for the gravitational torque effect (determined during the passive joint rotation driven by the dynamometer) according to Kellis & Baltzopoulos (33), and finally normalized to the mass of the subjects (e.g., expressed in Nm/kg).

**Figure 1:**
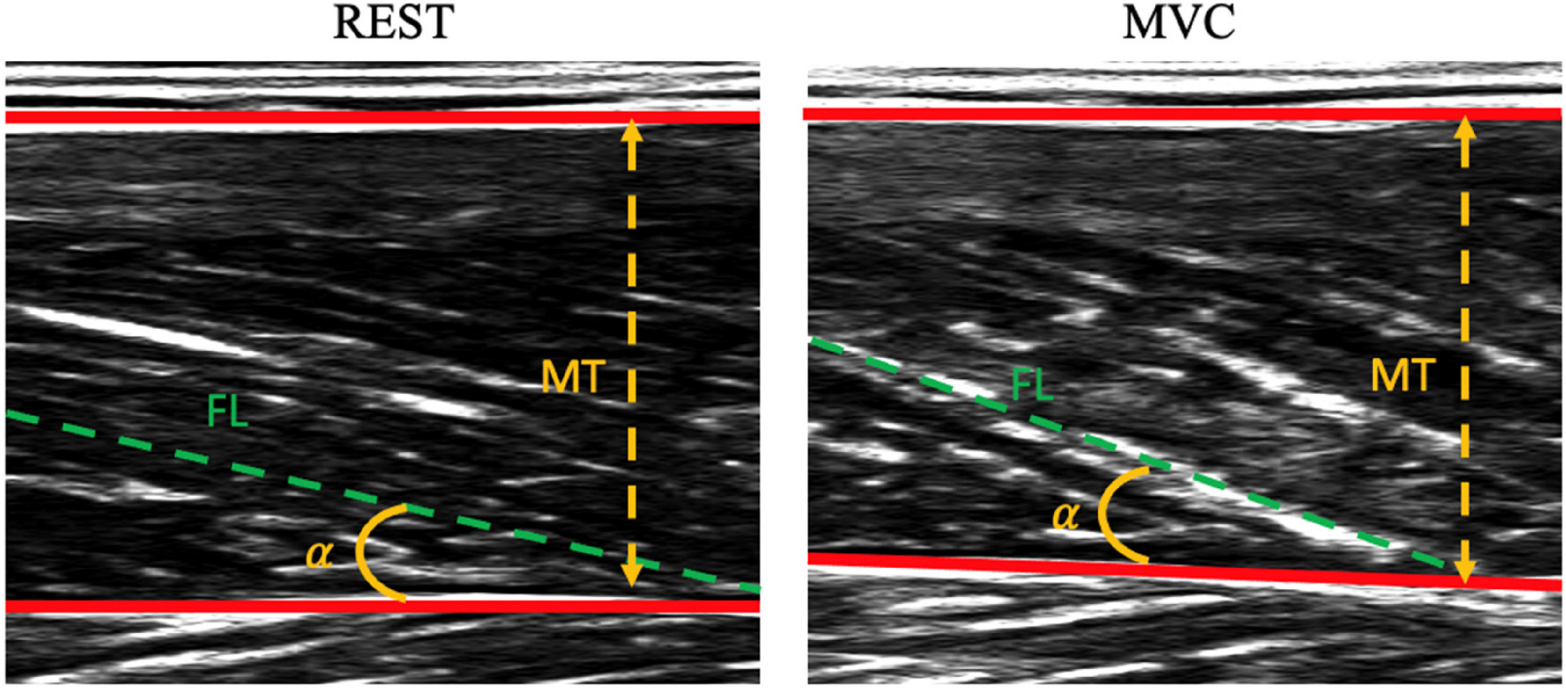
Representative ultrasound images of vastus lateralis at rest (panel on the left) and during contraction (panel on the right). Data refer to a patient with Parkinson's disease. MT, muscle thickness; *α*, pennation angle (PA); FL, fascicle length.

Ultrasound measurements during contraction were performed during the MVCs: on each ultrasound image, two points were manually digitalized on the superficial aponeurosis, two on the deep aponeurosis and two on a muscle fascicle through a Matlab custom script. The length of the muscle fascicles (FL) was defined as the distance along the fascicles between the deep and superficial aponeuroses; pennation angle (PA) was defined as the angle between the collagenous tissue and the deep aponeurosis; muscle thickness (MT) was defined as the perpendicular distance from the deep to the superficial aponeurosis in the middle of the field of view (34, 35) (see Figure 1). Since the VL fascicles could be longer than the field of view of the ultrasound probe, a linear extrapolation of the fascicle length was performed, as previously proposed in the literature (e.g., (22, 36–38). Representative ultrasound measurements were taken as a mean of three digitalized images. Changes in fascicle length (ΔFL), muscle thickness (ΔMT), and pennation angle (ΔPA) were calculated as the differences between the values determined during contraction and in the rest condition.

The values of torque at plateau (registered in the three MVCs at each knee joint angle), the corresponding values of fascicle length (FL at torque plateau) and the knee joint angles (obtained from the video analysis) were averaged and used to determine the torque-angle and T-L relationships. These relationships were then fitted with second-order polynomial functions based on which we calculated: (i) the maximum voluntary torque (*T*_max_) as the apex of the T-L relationship, (ii) the optimal knee joint angle as the angle at which *T*_max_ occurs and (iii) the optimal fascicle length (*L*_opt_) as the fascicle length at which *T*_max_ occurs.

Regarding the T-V relationship, the values of (peak) torque were calculated within the iso-velocity phase (during each isokinetic contraction); the acceleration and deceleration phases were thus excluded from data analysis [e.g., (39, 40)] (see Supplementary Figure S2).

Peak torque values were averaged among trials at the same angular velocity and used to reconstruct the T-V relationship (the values of angular velocity being determined by the Cybex software). Maximal angular velocity was determined as the intercept value on the abscissa of the T-V curve (fitted with a linear relationship).

Finally, the mechanical power values were calculated as the product between torque and angular velocity. The P-V curve was obtained by fitting the power and angular velocity data with a second-order polynomial function (41). Maximum mechanical power was calculated as the apex of the P-angular velocity curve. Power data are reported normalized for body mass (e.g., expressed in W/kg).

### Statistics

Data normality was assessed using a Shapiro-Wilks test. A one-way ANOVA was used to investigate differences in physical activity levels and anthropometric characteristics.

To accentuate the effect of the pathology, a one-way ANOVA was employed to delineate significant differences among the more affected limb of PD patients, and the CR and CY group in optimal knee angle, *L*_opt_, *T*_max_, *V*_max_ and *P*_max_. A two-way ANOVA was used to compare the groups cited above at the different knee angular positions or angular velocities to evaluate possible differences in specific portions of the T - L, T -V and P -V relationships. A *post hoc* Tukey test (with Bonferroni correction) was used. A paired *t*-test was then utilized to assess differences between PD patients' more affected and less affected limb.

Correlations between variables were calculated according to the Pearson correlation coefficient. In case the normality of the data was not verified, a Kruskal-Wallis test was performed and Spearman correlation coefficients were determined instead. Complete data are reported for 11 PD patients, 9 elderly controls and 10 young controls; due to the revised sample size, the *α* level was adjusted, as suggested by Gómez-de-Mariscal et al. (42). *A-posteriori* sample size analysis with the actual sample size (30 participants) indicated a *p*-value of 0.09; in order to reduce the Type1 error, the *p*-value was finally set as 0.08.

Statistical analysis was performed using Jamovi (v2.4.11) and SPSS 23 (IBM Corp., Armonk, NY, USA).

## Results

One PD patient, 7 elderly controls and 4 young controls were excluded from the final cohorts since they were not able to complete the experimental protocol or to modulate force in either the isometric and/or isokinetic trials; in these cases, the *R*^2^ of the T-L or T-V relationship was rather low (ranging from 0.40 to 0.57) whereas *R*^2^ was higher than 0.85 in all other cases/participants. Complete data are, thus, reported for 11 PD patients, 9 elderly controls and 10 young controls. As shown in Table 1, no differences were observed among groups regarding body mass and stature, as well as in physical activity level and cognitive function; according to the IPAQ scores, all participants were physically active. In patients, the UPDRS score and the H&Y score indicate a mild to moderate stage of the pathology (Table 2).

**Table 1:** Anthropometric characteristics, level of physical activity (IPAQ), cognitive status (MMSE), UPDRS-motor scale score, and H&Y score.

	Age (years)	Body mass (kg)	Stature (cm)	IPAQ (MET/week)	MMSE score	UPDRS score	H&Y score	Disease duration (years)
CY (*n* = 10; 4F/6M)	25.1 ± 1.73*	74.9 ± 14.9	176.2 ± 10.2	2,978 ± 1,657	29.4 ± 0.52	–	–	–
CR (*n* = 9; 1F/8M)	68.3 ± 5.36	78.7 ± 10.1	173.4 ± 8.10	3,916 ± 1,856	28.6 ± 1.67	–	–	–
PD (*n* = 11; 2F/9M)	70.5 ± 5.28	78.4 ± 12.5	172.5 ± 8.18	3,493 ± 1,230	28.6 ± 1.43	27.3 ± 13.2	1.50 ± 0.65	6.1 ± 4.36

CY: young subjects; CR: elderly control subjects; PD: subjects with Parkinson's disease.

IPAQ, International Physical Activity Questionnaire; MMSE, mini mental state examination; UPDRS, Unified Parkinson's Disease Rating Scale; H&Y, Hoehn and Yahr scale.

* *p* < 0.001 between CR and CY.

**Table 2:** Characteristics of subjects with Parkinson's disease.

Gender (M/F)	Age (years)	Body mass (kg)	Stature (cm)	Disease duration (years)	H&Y score	UPDRS score	MMSE score	Medication	Side of appearance of the first motor symptom
M	77	90	176	5	1	36	29	Madopar, Sinemed	Left
M	70	82	165	5	1	14	30	Madopar	Right
M	75	76	183	15	1	22	30	Mirapixin, Sinemed, Oxyen, Sirio	Right
M	65	67.5	178	2	1.5	31.5	27	Sinemed, Rantal	Right
M	73	72	180	7	1.5	20.5	30	Sinemed	Left
M	65	85	179	2	0.5	9	28	Madopar, Sinemed	Right
M	80	62	168	12	2	46	29	Mirapixin, Sinemed	Left
F	68	60	156	6	3	52	29	Sinemed, Mirapixin, Sirio	Left
F	66	78	165	0.2	1	21	30	None	Left
M	72	100	176	5	2	28	27	Sinemed, Ropinirol	Left
M	65	90	172	8	1.5	19	26	Sinemed, Requir	Right

Results are presented by considering the effect of ageing (CY vs. CR), pathology (CR vs. PDA) and the intra-subject effect of pathology (PDNA vs. PDA). In tables, data are reported as mean and standard deviation; in figures, data are reported as mean and standard error.

### T-Angle, T-L, T-V and P-V relationships

**Figure 2:**
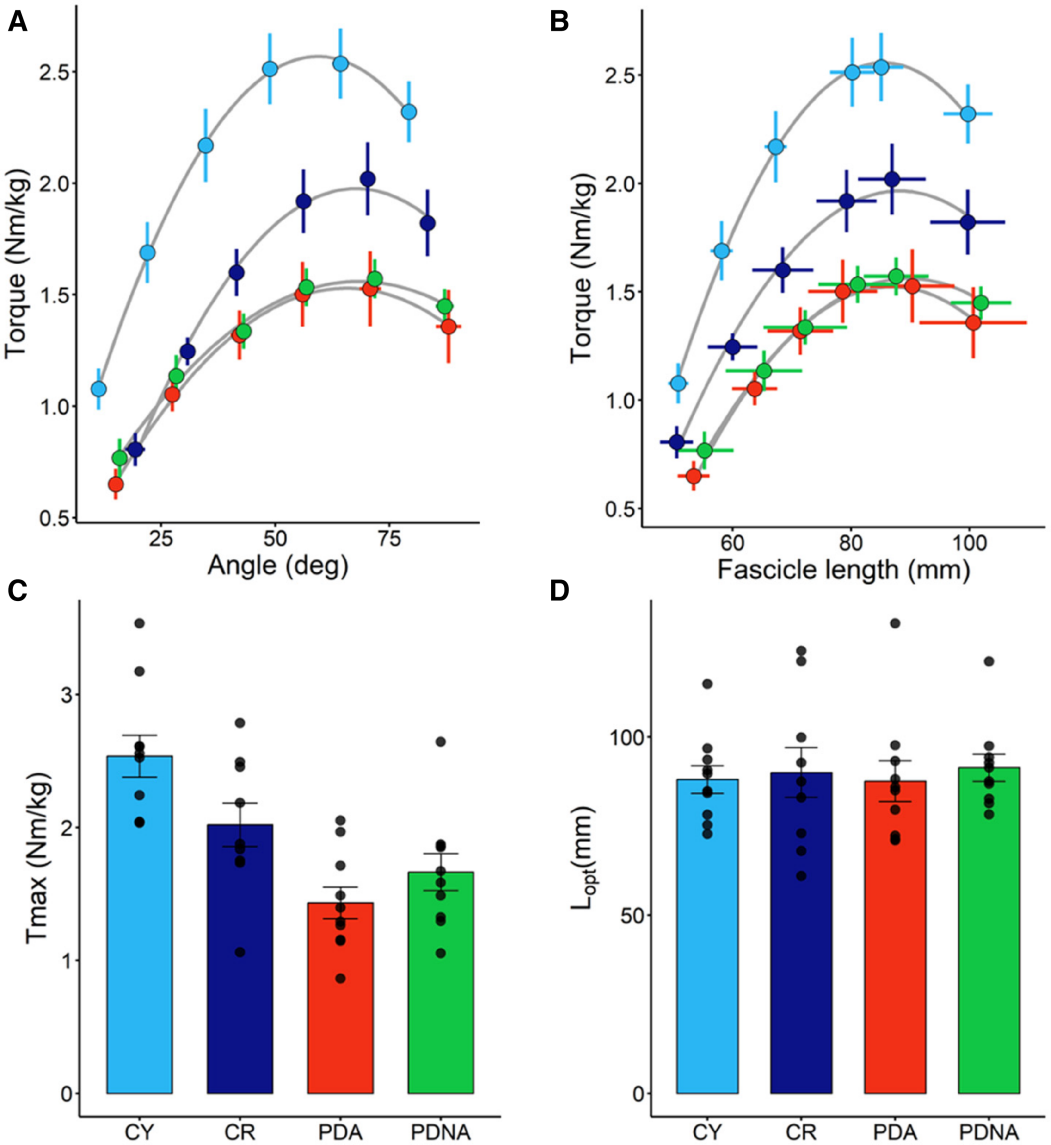
Torque-angle (panel **A**) and torque-fascicle length (panel **B**) relationships (fascicle data refer to VL); data points are means ± SE. The optimal length of the VL fascicles (*L*_opt_) and maximal torque (*T*_max_), as calculated from the torque-fascicle length relationship, are reported in panels **D** and **C**, respectively; data points represent individual data (means ± SE are represented as well). Light blue and dark blue data refer to control groups (young and middle-aged, respectively: CY and CR); red and green data refer to PD patients (more affected and less affected limb, respectively: PDA and PDNA). No differences were observed in *L*_opt_ among groups. For *T*_max_: CY vs. CR = 0.037; CY vs. PDA <0.001; CR vs. PDA = 0.031.

In Figure 2 the torque-fascicle length and the torque-angle relationships for the three groups are reported. *T*_max_ was higher in CY compared to CR (*p* = 0.037). Significant differences were also observed between CY and PDA (*P* < 0.001) and CR and PDA (*P* = 0.031). No significant effect of ageing or pathology was observed in optimal knee joint angle (CY vs. CR: *p* = 0.789; CR vs. PDA: *p* = 0.703) or optimal fascicle length (CY vs. CR: *p* = 0.879; CR vs. PDA: *p* = 0.632).

No significant differences were observed between PDA and PDNA in optimal fascicle length (*p* = 0.399), optimal knee joint angle (*p* = 0.767) and *T*_max_ (*p* = 0.153).

**Figure 3:**
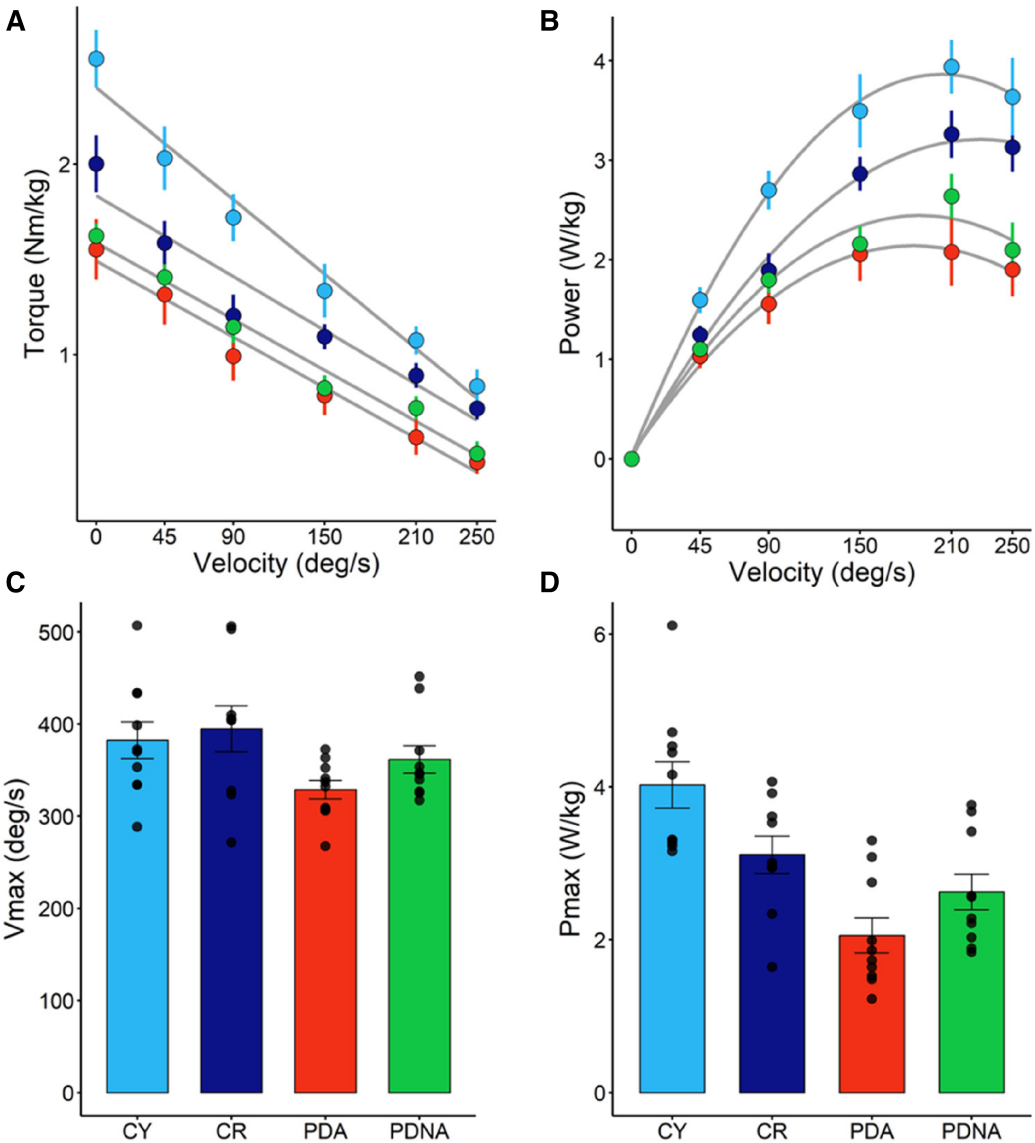
Torque-angular velocity (panel **A**) and power-angular velocity (panel **B**) relationships; data points are means ± SE. Maximal angular speed (*V*_max_) and maximal power output (*P*_max_), as calculated from the T-V and P-V relationships, are reported in panels **C** and **D**, respectively; data points represent individual data (means ± SE are represented as well). Light blue and dark blue data refer to control groups (young and middle-aged, respectively: CY and CR); red and green data refer to PD patients (more affected and less affected limb, respectively: PDA and PDNA). No differences were observed in *V*_max_ among groups. For *P*_max_: CY vs. CR = 0.05; CY vs. PDA <0.001; CR vs. PDA = 0.023; PDA vs. PDNA = 0.061.

In Figure 3 the T-V and P-V relationships for the three groups are reported. No significant effect of ageing or pathology in maximum angular velocity (*V*_max_) among groups (CY vs. CR = 0.845; CR vs. PDA = 0.153) was observed. Significant differences were observed in *P*_max_: CY showed higher values of *P*_max_ compared to CR (*p* = 0.05) and PDA (*p* < 0.001), while CR exhibited higher values of *P*_max_ compared to PDA (*P* = 0.023).

No significant differences were observed between PDA and PDNA in *V*_max_ (*p* = 0.485), but a significant difference was observed between PDA and PDNA in *P*_max_ (*P* = 0.064).

### Ultrasound measurement

**Figure 4:**
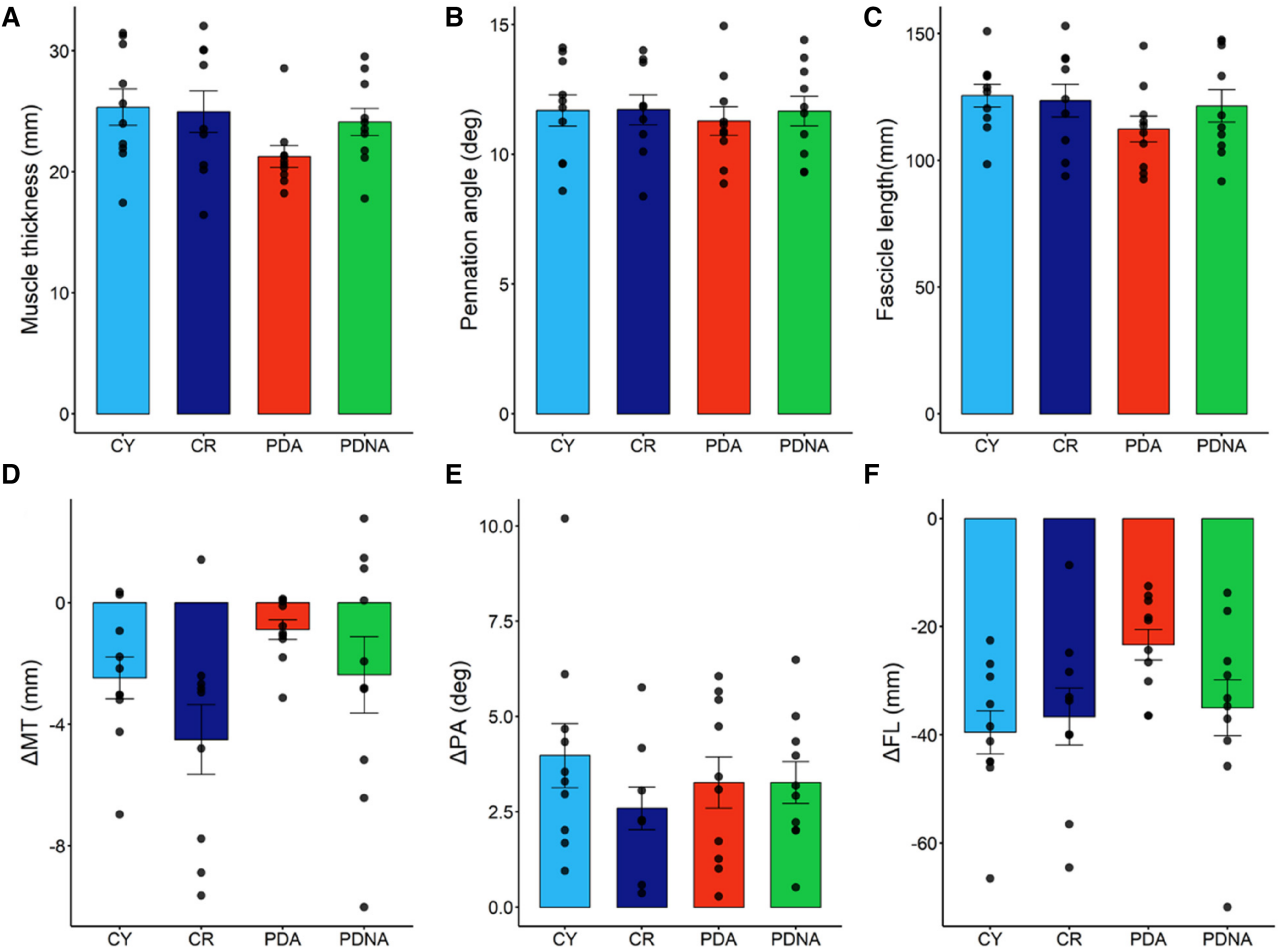
Fascicle length, muscle thickness, and pennation angle at rest (panels **A**–**C**, respectively). Dynamic changes (contraction-rest) are reported in panels **D**–**F**, respectively: fascicles shorten, muscle thickness decreases, and pennation angle increases. Data points represent individual data (means ± SE are represented as well). Light blue and dark blue data refer to control groups (young and middle-aged, respectively: CY and CR); red and green data refer to PD patients (more affected and less affected limb, respectively: PDA and PDNA). No differences were observed in ΔPA among groups. For ΔMT: CR vs. PDA = 0.022; for ΔFL: CY vs. CR = 0.025; CR vs. PDA = 0.069; PDA vs. PDNA = 0.077.

Data of fascicle length, pennation angle and muscle thickness at rest, and their changes from force onset till plateau during the MVCs, are reported in Figure 4.

Muscle thickness, pennation angle and fascicle length at rest showed no significant differences among groups. Changes in muscle thickness showed no significant differences between CY and CR (*p* = 0.285), while a significant effect of pathology was observed (CR vs. PDA: *p* = 0.022); the smallest changes were observed in PDA. No differences between PDA and PDNA were appreciated (*p* = 0.508).

Pennation angle was not affected by ageing (CY vs. CR: *p* = 0.110) or pathology (CR vs. PDA: *p* = 0.431) and no differences between PDA and PDNA were appreciated (*p* = 0.901).

Significant differences were observed in fascicle length as a function of age (CY vs. CR: *p* = 0.025) and pathology (CR vs. PDA: *p* = 0.069), as well as between PDA and PDNA (*p* = 0.077); the smallest changes were observed in PDA.

### Correlations between parameters

No significant correlations were observed between architectural parameters (at rest or changes) and *T*_max_, *V*_max_ or *P*_max_.

Moreover, no significant correlations were appreciated between each of the investigated parameters and the UPDRS and H&Y scores.

## Discussion

This study aimed to determine the effects of Parkinson's disease on muscle's mechanical output (T-L, T-V and P-V relationships) and its determinants (muscle geometry). For this reason, we recruited three groups of participants, matched for physical activity, to better distinguish the effect of ageing from the impairment imposed by the pathology itself. We observed an effect of age and pathology on maximal isometric force and maximal mechanical power: elderly participants had lower values of force and power compared to their younger counterparts, and Parkinson's disease exacerbated the loss of function (by comparing PD patients with healthy subjects of the same age). In addition, we observed differences in maximum mechanical power between the more and the less-affected side in PD patients. Last but not least, while muscle geometry at rest was similar among groups, significant differences were observed when investigating dynamic muscle shape changes in these populations.

Muscle weakness is one of the more critical factors that characterise functional disability in elderly people as well as in Parkinson's patients. Indeed, a decrease of muscle strength as a function of age is well established in the literature (see (43, 44). Our data support these findings, showing a downward shift of the T-L and T-V curves in elderly subjects compared to the young control group. We also observed a further reduction in *T*_max_ in people with Parkinson's disease, suggesting that maximum isometric torque, calculated as the apex of the T-L curve, could be a useful indicator of functional disability in this population.

Maintaining the ability to generate (and control) high-velocity movements is quite challenging in the elderly and, more so, in PD patients. The difficulty in generating high-speed movements could lead to functional disability and could affect the patient's quality of life (e.g., increasing the risk of falls) (45). Our data showed no significant differences among groups in V_max_ (maximum knee joint angular velocity); since muscle fascicle shortening velocity and knee angular velocity are likely correlated (46, 47), these data suggest that maximum contraction speed is preserved (in our experimental cohorts) regardless of age and pathology. Other studies showed a significant reduction in *V*_max_ as a function of age, but with differences that became evident after the 7th decade (2, 43). For example, Alcazar and colleagues (43) observed a non-significant difference in *V*_max_ (angular velocity) of the quadriceps muscles between young and middle-aged (40–60 years) subjects. The CR and PD participants of this study had an average age of 71 and 68 years, respectively, and, more importantly, were matched for physical activity, that could counteract the loss of function that occurs with age. To our knowledge, this is the first study that investigates the effects of PD on *V*_max_ and our data indicate that an active lifestyle could (at least) partially counteract the loss of *V*_max_ in this population.

Muscle power is one of the most important indices of functional capacity of an individual (5). In accordance with the literature, we observed an age-related decline in *P*_max_ (43, 48), which essentially reflects the loss in *T*_max_. Furthermore, our data indicate a further decrease in *P*_max_ in PD patients, suggesting that this variable could be another important indicator (along with *T*_max_) for investigating the functional capacity of this population. In this regard, it is important to note that *P*_max_ was the only variable able to identify differences between the less and the more-affected side in PD patients; the small differences between PDA and PDNA in *V*_max_ and *T*_max_ “sum up” and became significant when power, and hence *P*_max_, is calculated.

Optimal knee joint angle and optimal fascicle length were unaffected by age and pathology: the T-L and T-A curves showed a down-shift without any significant change in the fascicle's operating range. These findings could be primarily attributed to the similar values of muscle geometry in our groups at rest. Indeed, fascicle length and optimal knee joint angle depend on the operating length (49): increasing fiber length increases the absolute muscle active operating length as well as the knee joint range of motion. Moreover, a longer fascicle length is also related to higher values of maximum shortening velocity (50).

Previous studies did not show any difference in architectural parameters at rest between PD patients and age and physically-matched controls [e.g., (13, 15)], and this was also observed in the present study, reinforcing the idea that an active lifestyle is a key factor in preserving muscle structure in this population. However, significant differences were observed between PD patients and the other groups in terms of dynamic muscle changes (from rest to contraction). Indeed, the smaller changes in thickness and fascicle length (from rest to contraction) were observed in the vastus lateralis of PD patients (affected side). Similar results were reported by Monte et al. (13) during explosive contractions, where the authors indicate that reduced muscle's capability to change in shape in PD patients is associated with an increase in their muscle-tendon unit stiffness. The differences in muscle fascicle behavior observed in this study between PD patients and the other cohorts could, thus, be related to hypertonia of the musculoskeletal system (12) (or to other mechanical factors that could not be appreciated with a simple geometrical analysis at rest). Hence, these data underline, again (13), that the analysis of muscle and fascicle behaviour during contraction could provide further insight into the mechanical alteration imposed by PD, compared to standard imaging techniques at rest.

## Limitations and further considerations

The present study assessed knee extension muscle function, so these findings apply to this muscle action only. Evaluation of other muscles, such as the hip and plantar flexor muscles, cannot be performed with sufficient methodological rigor in the elderly/patients (e.g., for the difficulty of remaining in a prone lying position). Hence, even if other lower-limb muscles are more compromised in PD (51, 52), testing the knee extensors represents the best solution to evaluate muscle's mechanical alterations in these patients.

In this study, we did not investigate the co-activation of the antagonist muscles, but muscle co-activation might be altered in Parkinson's patients [e.g., (53–55)] owing the deterioration of motor control. Activation in all muscles that might act as antagonists is worth being assessed in future studies to better elucidate their contribution to maximum torque/power capacity.

Although we corrected the *p*-value for our sample size, our cohorts are limited in number (and further studies with larger groups should be performed). To note, we excluded several subjects for poor-quality data not related to data collection or analysis, but to the subject inability to correctly perform the assigned task. Thus, it is suggested, with these patients (and with elderly subjects in more general terms), to perform several familiarization sessions and to utilize real-time feedback to control the data quality during data collection.

In the present study, all participants were tested during the medication “on” condition of the dopaminergic treatment. Levodopa alleviates rigidity, rest tremors, and bradykinesia but could also affect EMG activity, force production and passive muscle tone in these patients (56–59). The “increase” in muscle tone during the “off” phase of the pharmacological treatment is expected to exacerbate the impairment in muscle's contractile capacity; on the other hand, the symptoms that are alleviated by levodopa could interfere with the evaluation of the muscle's contractile capacity.

Different fitting procedures (for the T-V relationship) provide different estimates of *V*_max_ (e.g., (60). We tested both the linear fitting and the linear-hyperbolic fitting (as proposed by (41), but no significant differences were appreciated in *V*_max_ comparing these two modalities. Hence, we opted for the linear fitting, due to its simplicity.

Due to the age-dependent reduction in the quality of ultrasound images (61) and the low sampling rate it was not possible to obtain high-quality data during the dynamic contraction phase in the MCVs; for this reason, only values of MT, FL and PA at rest and during contraction (at steady state) are reported in this study.

Last but not least, we focused on the peripheral factors known to affect muscle function (dynamic muscle shape changes), but the differences we observed could also be attributed to neural/nervous factors not investigated in this study.

## Conclusions

The present study revealed significant differences between PD patients and age and physically matched control groups in terms of maximum isometric torque (calculated as the apex of the T-L curve) and maximum mechanical power (apex of the P-V curve), while the maximum angular velocity of the knee extensor muscles was not affected by the pathology. Among the mechanical variables investigated, mechanical power was able to identify differences between the less and the more affected side of the body in PD patients, suggesting that this parameter could be useful for clinical evaluation in these patients. The observed results cannot be explained by differences in muscle geometry at rest, but rather by the muscle capacity to change in shape during contraction, that is affected in PD patients.

## Data Availability

The raw data supporting the conclusions of this article will be made available by the authors, without undue reservation.
