# Supplementary material for: Characterization of the vastus lateralis torque-length, and knee extensors torque-velocity and power-velocity relationships in people with Parkinson's disease
Source: Front Sports Act Living. 2024 Apr 25;6:1380864. doi: 10.3389/fspor.2024.1380864 (PMC11079174; doi:10.3389/fspor.2024.1380864)

**Characterization of the vastus lateralis Torque-Length, and knee extensors Torque-Angle, Torque-Velocity and Power-Velocity relationships in people with Parkinson’s disease**

**Riccardo Magris, Francesca Nardello, Federica Bombieri, Andrea Monte, Paola Zamparo**

*Department of Neurosciences, Biomedicine and Movement Sciences, University of Verona, Verona, Italy*

**Figure S1:** Experimental procedures. The participants were secured on a dynamometer using a trunk and pelvic strap. The rotational axis of the dynamometer was carefully aligned with the axis of rotation of the knee joint during a maximal contraction at 60° knee flexion. A standardised warm-up for each contraction type was conducted to familiarise the subjects with the task (maximal voluntary contractions, MCVs, and isokinetic knee extensions).


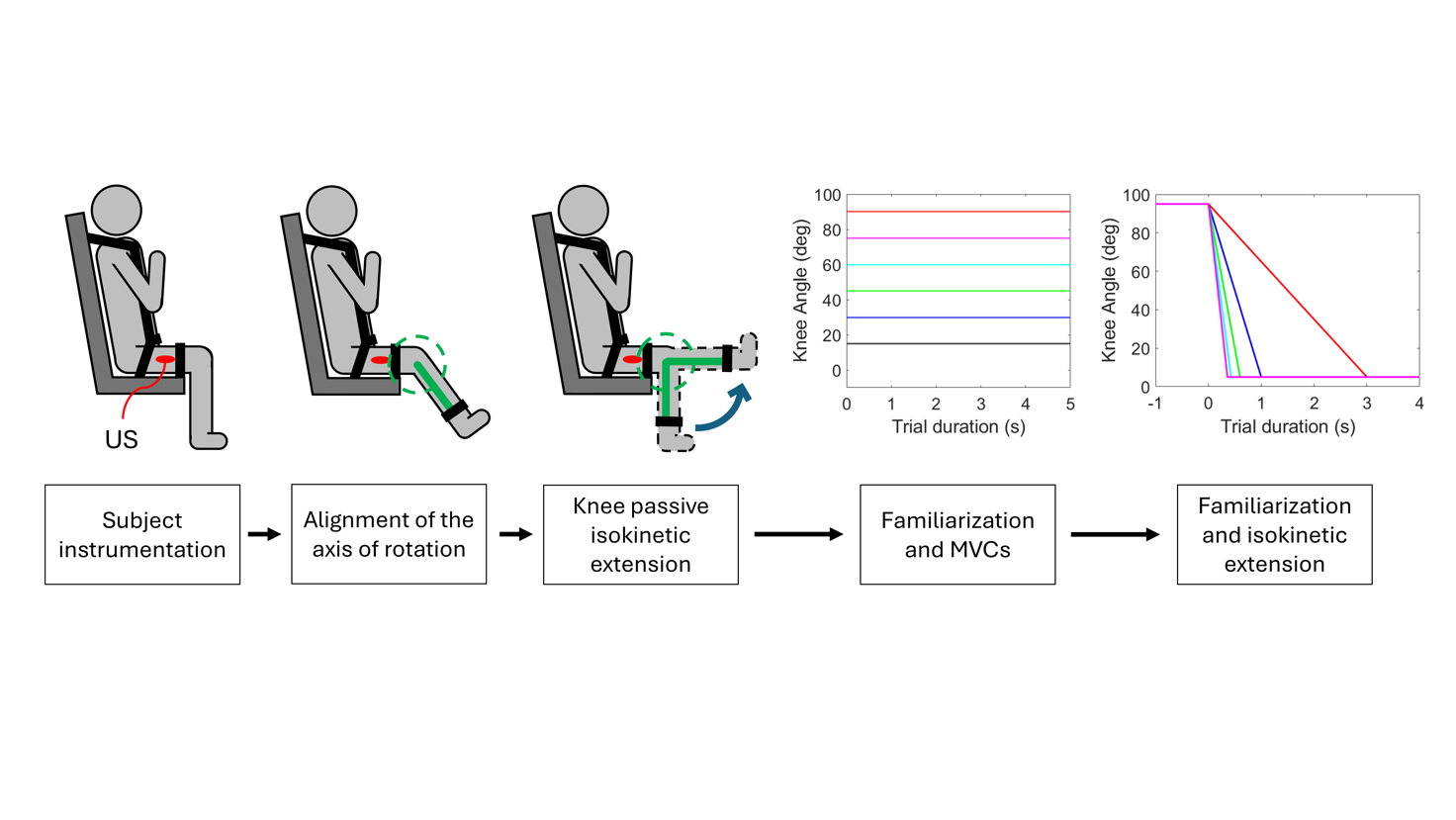


**Figure S2**: Dynamometer angular velocity (blue line) and knee joint torque (red line) as a function of contraction duration at three angular speeds (top panels: 45°/s; central panels: 150°/s; bottom panels: 250°/s). Vertical dashed (black) lines refer to the start and the end of the isokinetic phase. The values of (peak) torque were calculated within the iso-velocity phase; the acceleration and deceleration phases were thus excluded from data analysis. CY: young control subject; PD: patient (more affected limb).


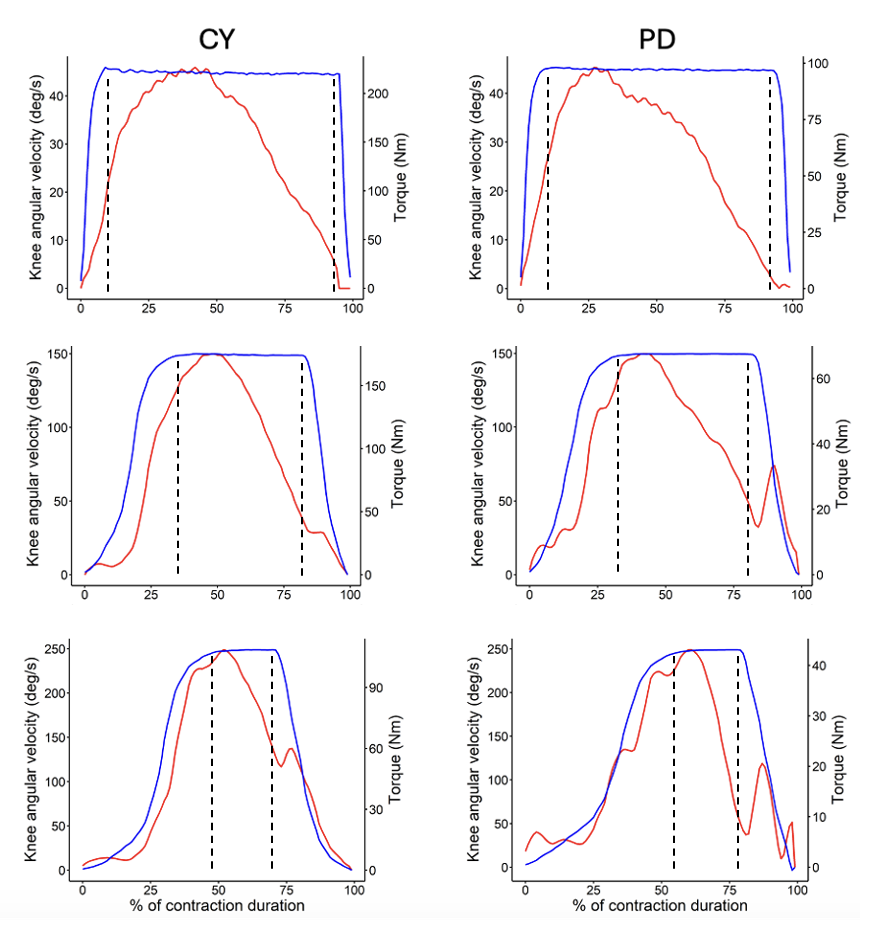

Supplement: Supplementary file 1 [file Table1.docx]
